# High glucose promotes pancreatic cancer cells to escape from immune surveillance via AMPK-Bmi1-GATA2-MICA/B pathway

**DOI:** 10.1186/s13046-019-1209-9

**Published:** 2019-05-14

**Authors:** Qingke Duan, Hehe Li, Chenggang Gao, Hengqiang Zhao, Shihong Wu, Heshui Wu, Chunyou Wang, Qiang Shen, Tao Yin

**Affiliations:** 1 Department of Pancreatic Surgery, Union Hospital, Tongji Medical College, Huazhong University of Science and Technology, Wuhan, 430022 China; 2 Department of Clinical Cancer Prevention, The University of Texas MD Anderson Cancer Center, Houston, TX 77030 USA

**Keywords:** Pancreatic cancer, NK cells, High glucose, MICA/B, Immune surveillance

## Abstract

**Background:**

Modulation of cell surface expression of MHC class I chain-related protein A/B (MICA/B) has been proven to be one of the mechanisms by which tumor cells escape from NK cell-mediated killing. Abnormal metabolic condition, such as high glucose, may create a cellular stress milieu to induce immune dysfunction. Hyperglycemia is frequently presented in the majority of pancreatic cancer patients and is associated with poor prognosis. In this study, we aimed to detect the effects of high glucose on NK cell-mediated killing on pancreatic cancer cells through reduction of MICA/B expression.

**Methods:**

The lysis of NK cells on pancreatic cancer cells were compared at different glucose concentrations through lactate dehydrogenase release assay. Then, qPCR, Western Blot, Flow cytometry and Immunofluorescence were used to identify the effect of high glucose on expression of MICA/B, Bmi1, GATA2, phosphorylated AMPK to explore the underlying mechanisms in the process. Moreover, an animal model with diabetes mellitus was established to explore the role of high glucose on NK cell-mediated cytotoxicity on pancreatic cancer in vivo.

**Results:**

In our study, high glucose protects pancreatic cancer from NK cell-mediated killing through suppressing MICA/B expression. Bmi1, a polycomb group (PcG) protein, was found to be up-regulated by high glucose, and mediated the inhibition of MICA/B expression through promoting GATA2 in pancreatic cancer. Moreover, high glucose inhibited AMP-activated protein kinase signaling, leading to high expression of Bmi1.

**Conclusion:**

Our findings identify that high glucose may promote the immune escape of pancreatic cancer cells under hyperglycemic tumor microenvironment. In this process, constitutive activation of AMPK-Bmi1-GATA2 axis could mediate MICA/B inhibition, which may serve as a therapeutic target for further intervention of pancreatic cancer immune evasion.

**Electronic supplementary material:**

The online version of this article (10.1186/s13046-019-1209-9) contains supplementary material, which is available to authorized users.

## Background

Pancreatic cancer is a highly malignant tumor of digestive system and its incidence is increasing rapidly in recent years. The early diagnosis is still very difficult for pancreatic cancer patients. Most pancreatic cancer patients are diagnosed with advanced stage and the prognosis is poor [1]. For the patients suffering from pancreatic cancer, the 5-year relative survival rate is less than 8% [2]. Therapy failure in most pancreatic cancer patients is mainly due to distant metastasis before surgical operation and limited efficiency of chemotherapy or radiation therapy [3]. It is urgently necessary to elucidate the underlying mechanisms of pancreatic cancer progression and develop effective therapies.

Clinical studies have proven that pancreatic cancer has close relation with hyperglycemia. Diabetes mellitus have been proved to increase the incidence of pancreatic cancer compared with non-diabetes population [4]. The mortality of pancreatic cancer patients associated with diabetes mellitus is significantly higher than those without diabetes [5]. Moreover, pancreatic cancer patients with diabetes mellitus frequently showed larger tumors and reduced median survival [6]. Unfortunately, the definite role and molecular mechanisms of hyperglycemia in the progression of pancreatic cancer have not been clearly elucidated until now.

The immune system plays an important role in the development of pancreatic ductal adenocarcinoma. Unfortunately, the immune system seems imbalanced in pancreatic cancer patients, facilitating spontaneous cancer development [7]. Despite the presence of many immune cells in pancreatic cancer tissue, immune dysfunction is observed where the tumor microenvironment is immunosuppressive, leading to inhibited activation of immune effectors. Natural killer (NK) cells are vital components of innate immune system. NK cells can kill cancerous cells through recognizing the ligands expressed on the surface of tumor cells [8]. NK cells are recognized as the first line of defense against cancer [9], and have gained much attention in adoptive cancer immunotherapy. The killing effect of NK cells mainly relies on its activating receptors NKG2D, which can bind to NKG2D ligands (NKG2DLs) on target cells and mediate the cytotoxicity [10]. MHC class I chain related molecules A/B (MICA/B) is a highly glycosylated membrane protein, belonging to NKG2DL family [10]. As the ligand of NKG2D, MICA/B can activate NK cells specifically to induce immune killing. However, tumor cells can escape from immune surveillance mediated by NKG2D through shedding or weakening MHC class I chain related molecules (MIC) from the membranes of cancer cells [11].

In this study, we demonstrated that high glucose inhibited the cell surface expression of MICA/B on pancreatic cancer cells and weaken the cytotoxicity of NK cells on pancreatic cancer. Moreover, high glucose promoted the expression of polycomb protein Bmi1, which increased GATA2 and inhibited cell surface MICA/B expression. Bmi1 is a major component of Polycomb Repressor Complex 1 (PRC1) family, and was originally identified as an oncogene associated with the development of murine lymphoma [12]. In this study, we identified a novel role of Bmi1 in pancreatic cancer immune escape. Our results demonstrated a new pathway of AMPK-Bmi1-GATA2-MICA/B axis, which was activated under high glucose and shown to be essential for the immune escape of pancreatic cancer cells.

## Methods

### Cell culture

The pancreatic cancer cell lines, PANC-1 and SW1990, were obtained from ATCC, and were cultured in DMEM medium containing 10% fetal bovine serum and 100 U/ml penicillin/streptomycin mixture (Beyotime Biotechnology, Shanghai, China). NK cells were originally obtained from China Center for Type Culture Collection (CCTCC), and cultured in α-MEM containing 12.5% horse serum, 12.5% fetal bovine serum and 200 U/ml of recombinant human interleukin − 2 (rhIL-2). The cells were cultured in 37 °C with 5% CO2. The concentration of glucose was 5 mM for general cell culture and in order to simulate the high glucose environment, two levels of diabetogenic glucose concentration (15 mM and 25 mM) were chosen.

### Western blot analysis

After washing three times with PBS, total cell lysates were extracted with RIPA lysis buffer. Quantitation of proteins was performed using the BCA protein concentration kit (Beyotime Biotechnology, Shanghai, China) and 30 μg of each sample was used to SDS-PAGE electrophoresis and transferred to the PVDF membranes (Millipore, Billerica, MA, USA). The membranes were blocked in 5% non-fat milk for 1 h and incubated with primary antibody at 4 °C over night. After washing 3 times with TBST (10 min/ times), the membranes were incubated with second antibody at room temperature for 1 h. After washing another 3 times with TBST, they were visualized with enhanced chemiluminescence (Pierce, Thermo Fisher, Waltham, MA, USA). The primary antibodies against GAPDH (1:1000), Bmi1 (1:1000), p-AMPK (1:1000), and AMPK (1:1000) were purchased from CST (Cell Signaling Technology, Danvers, MA, USA). MICA/B (1:200) antibody was purchased from Santa Cruz (Santa Cruz Biotechnology, Texas, U.S.A.). GATA2 (1:1000) antibody was purchased from Abcam (Abcam, Cambridge, UK). GAPDH was used as the internal control. AMPK activator (A-769662) and AICAR were obtained from Selleck chemicals (Selleck.cn, Shanghai, China).

### Quantitative real-time PCR

After washed with PBS, the total RNA of the treated cells was extracted using TRIzol. cDNA was obtained by reverse transcription through the reaction of 1 μg RNA and PrimeScript™ RT Master Mix (Takara Bio, Shiga, Japan). qRT-PCR is obtained according to the SYBR Green PCR Kit (Takara Bio, Shiga, Japan). The results were analyzed according to 2^-ΔΔCT^, and GAPDH was used as control. Primer sequences for GAPDH, Bmi1, GATA2, MICA, and MICB were shown in Additional file 3: Table S1.

### ChIP

Chromatin immunoprecipitation (ChIP) was performed using anti-GATA2 antibody and EZ ChIP™ Chromatin Immunoprecipitation Kit (Millipore, Billerica, MA, USA), following the manufacturer’s protocol. The IgG was used as the internal control. After high glucose treatment, ChIP was performed by immunoprecipitation with IgG or GATA2 antibody. The bound DNA fragments were amplified with MICA/B promoter-specific primers. The PCR products were resolved by electrophoresis. Primer sequences for ChIP-qPCR were shown in Additional file 3: Table S1.

### Cell transfection

Bmi1 over-expression cDNA (pcDNA3.1-Bmi1) and empty vector cDNA (pcDNA3.1-NC) were designed and synthesized by GenePharma (Shanghai, China). The GATA2 siRNA/ Bmi1 siRNA and NC siRNA were designed and synthesized by Ribobio (Guangzhou, China). Lipofectamine™2000 (Invitrogen, California, U.S.A.) was used in the cell transfection according to manufacturer’s protocol. After transfection for 6 h, the medium was replaced to normal medium. The siRNA sequences and negative control siRNA sequences were shown in Additional file 4: Table S2.

### Flow cytometry analysis

After transfection for 48 h of Bmi1 plasmid or GATA2 siRNA/Bmi1 siRNA, the culture medium was discarded. The cells were digested with trypsin and centrifugated. After washing three times with PBS, they were made into monocellular suspension. Each test tube was given 10 μL MICA/B -PE antibody (R&D systems, Minnesota, U.S.A.) under the condition of dark light, and incubated for 30 min at 4 °C. After washing three times, the tubes were added with 200 μl PBS before analyzing. The results were obtained by flow cytometer analysis.

### Lactate dehydrogenase (LDH) release assay

The killing ability of NK cells was analyzed by LDH release assay according to manufacturer’s protocol (Beyotime Biotechnology, Shanghai, China). Briefly, the target cell is 10 thousand, and the effective target ratio is 2.5:1, 5:1, 10:1 and 20:1 in 96-well plates. LDH release assay was performed after incubation for 4 h in 37°C and 5% CO2. The killing activity of NK cells was calculated as following: the killing activity (%) = (OD experimental group - OD natural release)/(OD maximum release - OD natural release)*100%.

### Immunofluorescence

Pancreatic cancer cells were plated in 12-well plates with a density of 1 × 10^4^ cells/well. After washing with cold PBS, the cells were fixed with 4% paraformaldehyde in PBS for 15 min at room temperature. Then, they were permeabilized with 0.5% Triton-X, blocked with goat serum, incubated with Bmi1 primary antibody at 4 °C over night. After washing with PBS, the cells were incubated with fluorescent secondary antibody for 2 h at room temperature. Then the samples were stained with DAPI for 5 min and photographed with fluorescence microscopy.

### Immunohistochemistry (IHC) analysis

We investigated the association between Bmi1 and MICA/B expression in cancer tissue using tissue microarrays. Two arrays (same set) contained 30 cases of pancreatic cancer tissues (Outdo Biotech, Shanghai, China) were obtained. The first array was stained with anti-Bmi1 antibody (Cell Signaling Technology, Danvers, MA, USA) and the second array was stained with anti-MICA/B (Santa Cruz Biotechnology, Texas, U.S.A.) antibody using standard IHC protocol.

### Animal experiments

For the diabetic pancreatic cancer mice model, the 5-week male Balb/c athymic nude mice (Beijing Vital River Laboratory, Beijing, China) were randomly divided into Control, Hyperglycemia, Hyperglycemia + insulin, Control + NK, Hyperglycemia + NK, and Hyperglycemia + insulin + NK groups. For the diabetic groups, the mice were injected streptozocin (STZ) (Sigma, St. Louis, MO, USA) at a concentration of 175 mg/kg on Day 0 and Day 7, respectively and STZ was dissolved in cold sodium citrate buffer (pH = 4.5). Blood samples were taken from the tail vein and measured with SANNUO (Changsha, China). The mice with blood glucose > 300 mg/dL were included in our experiments (*n* = 5 in each group). After establishing the diabetes model, two groups were injected insulin (0.8 units/kg/day) to normalize blood glucose level. Then the six groups were all subcutaneously injected PANC-1 cells (3× 10^6^ /100 μL/mouse) in the right flank. One week after subcutaneous implantation, NK cells (10^5^/mouse) were injected into mice once a week for three weeks. The animals were sacrificed one week after the last NK cell injection. The tumor size was measured periodically and calculated by the formula 0.5 × length × width^2^. The expression levels of Bmi1, MICA/B and GATA2 were measured with IHC.

### Statistical analysis

The results were shown as mean ± SD. The Western blot results were analyzed by Image Lab 3.0 software (Bio-Rad, Hercules, CA, USA). Comparisons between the two treatments were evaluated using Student’s *t* test. Comparisons between multiple groups were performed with Two-way ANOVA analysis. The SPSS 21.0 software was used for statistical analysis and *P* < 0.05 was considered as statistically significant.

## Results

### High glucose reduces NK cell-mediated lysis of pancreatic cancer cells by inhibiting cell surface MICA/B expression

**Fig. 1:**
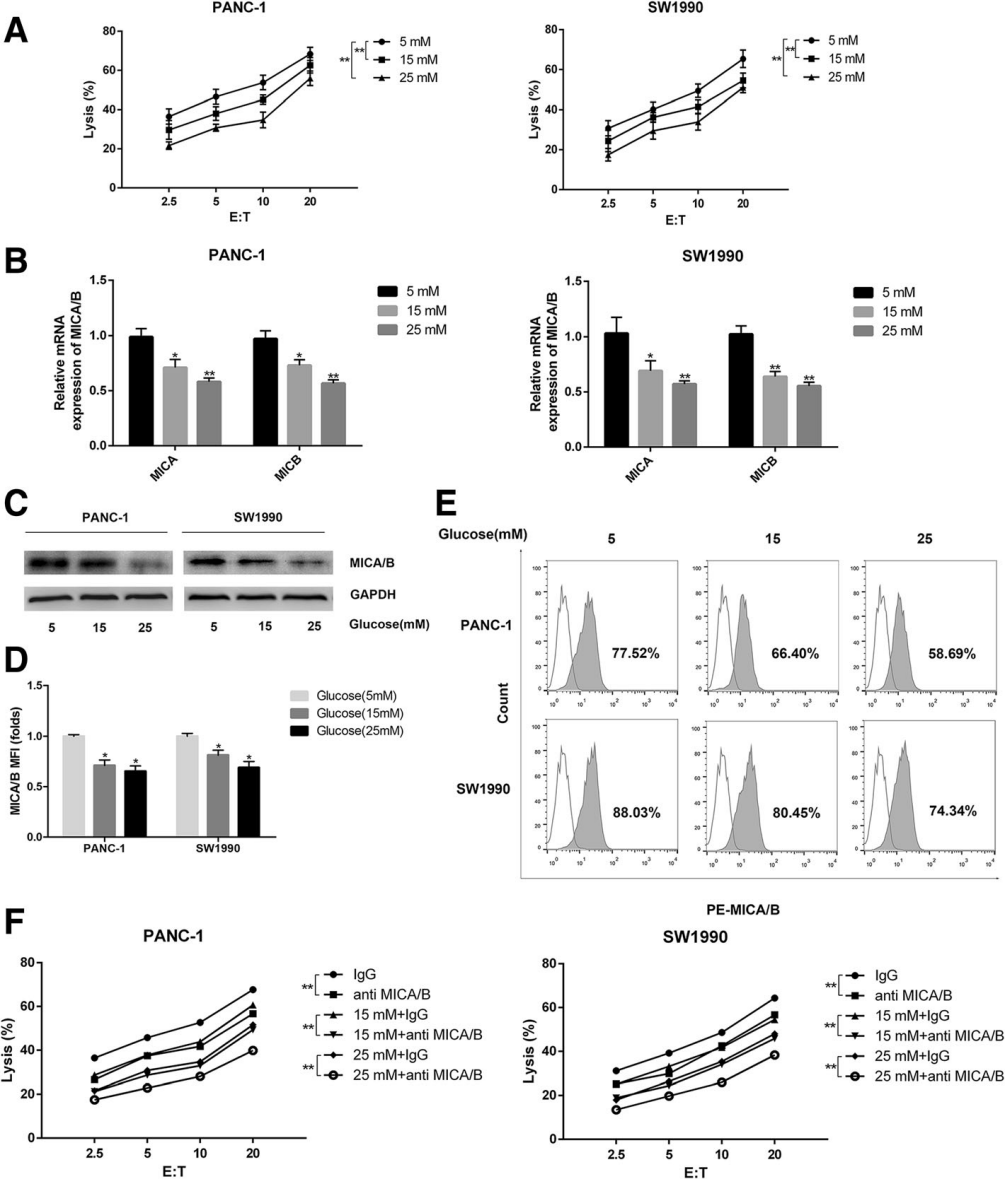
High glucose reduces NK cells^,^ killing effect on pancreatic cancer cells through suppressing cell surface MICA/B expression. The PANC-1 cells and SW1990 cells were treated with different concentrations of glucose (5 mM, 15 mM and 25 mM) for 24 h respectively. **a** The killing effects of NK cells on pancreatic cancer cells were detected by LDH release assay. E: T = Effector cells: target cells. **b-c** The cell surface expression of MICA/B was detected by qRT-PCR (**b**) and Western blot (**c**). **d** MFI (folds) of MICA/B detected by flow ctyometry was evaluated with a Student t test from three independent experiments. **e** Representative histograms of flow cytometry demonstrating MICA/B expression in pancreatic cell treated with different concentrations of glucose. **f** The killing effects of NK cells on pancreatic cancer cells in the presence of anti-MICA/B antibody treatment (displayed as mean value). The graphs shown were from three independently experiments. The data were from three independently repeated experiments. **P < 0.01; *P < 0.05

In order to investigate the effect of high glucose on the killing effect of NK cells on pancreatic cancer cells, we co-cultured NK cells (Effector) with two pancreatic cancer cell lines, PANC-1 and SW1990 (Target) in media with different concentrations of glucose. The killing effect was determined with a LDH release assay. In both cell lines, percentage of pancreatic cancer cells under lysis increased with the increasing of Effector to Target (E:T) ratio from 2.5 to 20 comparing with normal glucose group. These results showed that the killing effect of NK cells was reduced with increasing glucose concentrations in culture medium (Fig. 1a). MICA/B molecules are expressed in a variety of tumors, including breast, melanoma and hepatocellular cancers and are important NKG2D ligands [13, 14]. To determine whether MICA/B is involved in the decreased killing effect induced by high glucose, we detected the expression of MICA/B by qRT-PCR, Western blot and flow cytometry after high glucose treatment. Cell surface MICA/B expression was significantly decreased with increase of glucose concentrations both at mRNA and protein levels in PANC-1 and SW1990 cell lines (Fig. 1b-e). To further explore whether the decreased killing of NK cells was related with the changes of MICA/B expression in pancreatic cancer cells, we performed anti-MICA/B blocking experiments with specific antibody against MICA/B. Application of MICA/B antibody significantly blocked the killing effect in both tested pancreatic cancer cell lines (Fig. 1f).

### Bmi1 inhibits cell surface MICA/B expression and reduces the NK cell cytotoxicity on pancreatic cancer cells

**Fig. 2:**
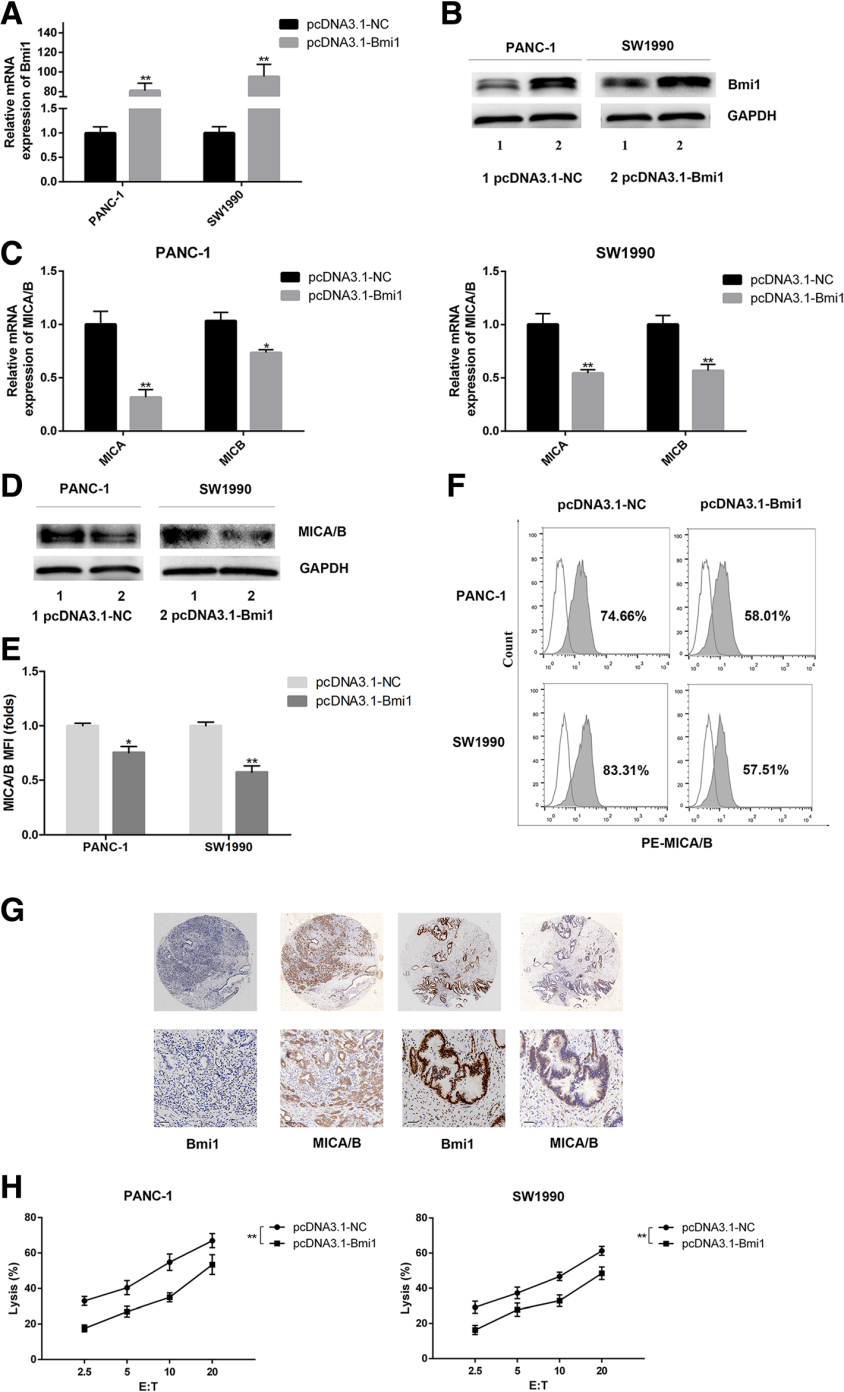
Bmi1 inhibits cell surface expression of MICA/B and blocks NK cell-mediated lysis in pancreatic cancer cells. **a-b** Expression of Bmi1 at mRNA and protein levels were detected by q-PCR (**a**) and Western blot (**b**) in PANC-1 and SW1990 cell lines after transfection of Bmi1 cDNA. **c**-**d** Expression of MICA/B at mRNA and protein levels were detected by q-PCR (**c**) and Western blot (**d**) after Bmi1 overexpression. **e** MFI (folds) of MICA/B detected by flow ctyometry was evaluated with a Student t test from three independent experiments. **f** Representative histograms of flow cytometry demonstrating MICA/B expression in pancreatic cell after Bmi1 overexpression. **g** Immunohistochemistry results reveal that cell surface MICA/B show low expression where Bmi1 is high expressed in pancreatic cancer tissues. **h** The killing effect of NK cells on pancreatic cancer cells overexpression Bmi1. The graphs show representative results from three independently repeated experiments. The data were from three independently repeated experiments. **P < 0.01; *P < 0.05

Previous study showed that endogenous expression of Bmi1 promotes invasion and progression in pancreatic cancer [15]. However, the role of Bmi1 in regulating immunity in pancreatic cancer was not defined. In this study, we determined the influence of Bmi1 on immunological characteristics of pancreatic cancer. Bmi1 overexpression was achieved by transfection of overexpression vector (Fig. 2a-b) into pancreatic cancer. Interestingly, Bmi1 overexpression caused reduced cell surface MICA/B expression, which was verified by qRT-PCR, Western blot and flow cytometry assay (Fig. 2c-f). Vice versa, after Bmi1 knockdown by siRNA, cell surface MICA/B expression was increased (Additional file 1: Figure S1). We further detected the correlation between Bmi1 and MICA/B in pancreatic cancer tissue using immunohistochemistry. We found that MICA/B expression was decreased where Bmi1 expression was high in pancreatic cancer tissues (total 30 cases, *P* < 0.01) and the representative results were shown in Fig. 2g. We further explored the effect of Bmi1 overexpression on NK cell cytotoxicity on pancreatic cancer cells. Pancreatic cancer cells with Bmi1 overexpression were co-cultured with NK cells, and the killing effect was detected using LDH release assay. We found that Bmi1 overexpression inhibited NK cell killing in both pancreatic cancer cell lines (Fig. 2h). On the other hand, the cytotoxicity of NK cells on pancreatic cancer cells increased when Bmi1 was inhibited with siRNA transfection (Additional file 1: Figure S1).

### High glucose reduces cell surface MICA/B expression by promoting Bmi1 expression

**Fig. 3:**
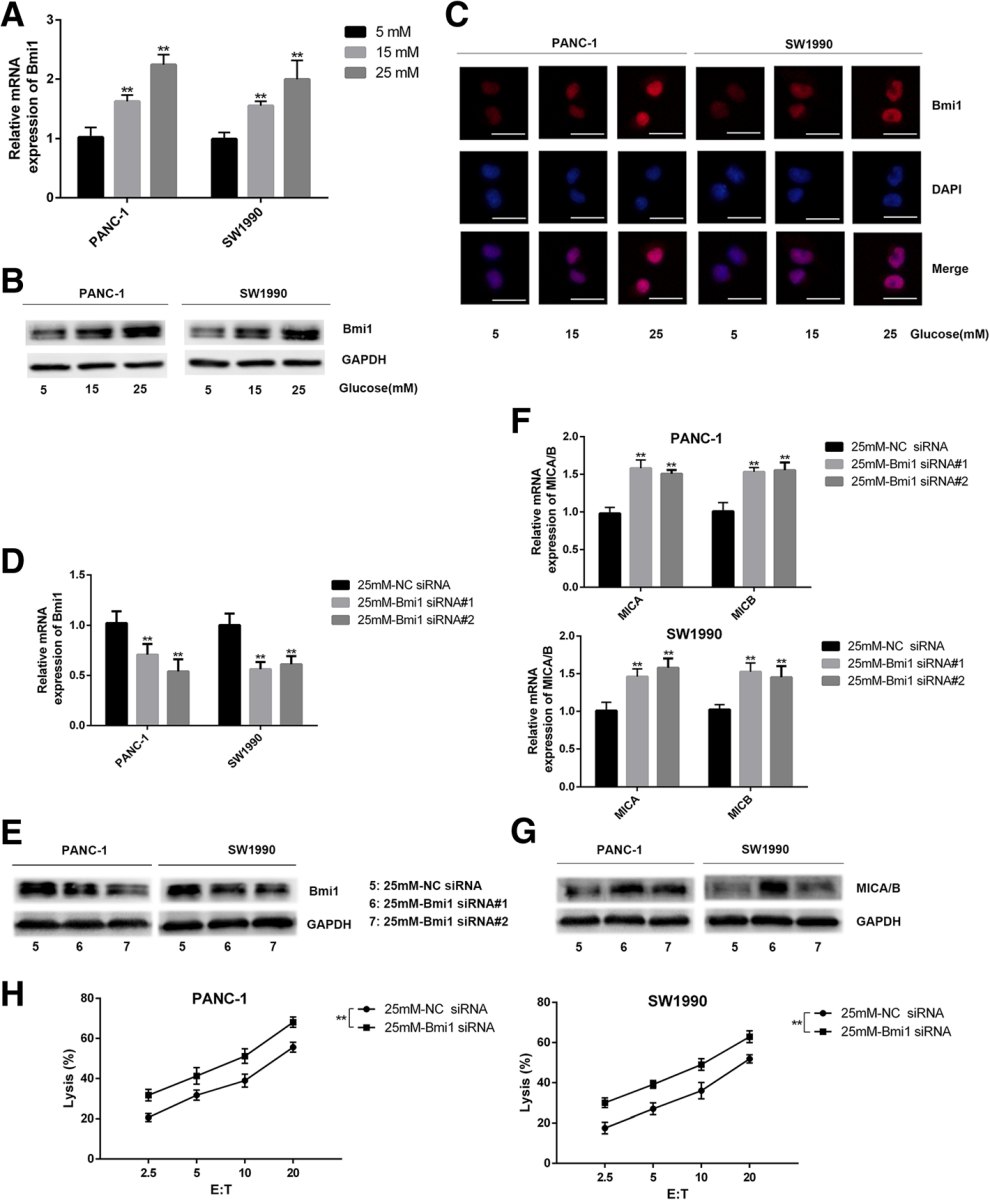
High glucose reduces the cell surface expression of MICA/B by promoting Bmi1 expression. **a-c** Expression of Bmi1 detected by qRT-PCR (**a**) Western blot (**b**) and immunofluorescence (**c**) in two pancreatic cancer cell lines treated with high glucose for 24 h. **d**-**e** Expression of Bmi1 detected by qRT-PCR (**d**) and Western blot (**e**) after transfection with Bmi1-siRNA under high glucose environment. **f**-**g** Cell surface expression of MICA/B at mRNA and protein levels detected by qRT-PCR (**f**) and Western blot (**g**) in both pancreatic cancer cell lines after Bmi1 knockdown under high glucose environment. **h** The killing effect of NK cells on pancreatic cancer cells after Bmi1 knockdown under high glucose environment. The graphs show representative results from three independently repeated experiments. The data were from three independently repeated experiments. Scale bar, 50 μm, **P < 0.01

We further explored the effect of high glucose on Bmi1 expression in pancreatic cancer cells. Bmi1 expression was detected by qRT-PCR, Western blot, and immunofluoresence in two cell lines with treatment of different concentrations of glucose. Our results showed that Bmi1 expression increased after high glucose treatment (Fig. 3a-c). To further explore the effect of Bmi1 on MICA/B expression under high glucose, the changes of MICA/B were detected in pancreatic cancer cells transfected with Bmi1 siRNA and control siRNA for 48 h under high glucose. We found that Bmi1 siRNA knockdown remarkably decreased Bmi1 expression at mRNA and protein levels in two pancreatic cancer cell lines in hyperglycemic medium. Correspondingly, MICA/B expression was increased after Bmi1 silence, which was verified by qRT-PCR, Western blot, and flow cytometry (Fig. 3d-g). Moreover, lysis of pancreatic cancer cells by NK cells increased under high glucose after Bmi1 knockdown with siRNA transfection (Fig. 3h).

### Bmi1 overexpression promotes GATA2 expression, which in turn specifically inhibits cell surface MICA/B expression

**Fig. 4:**
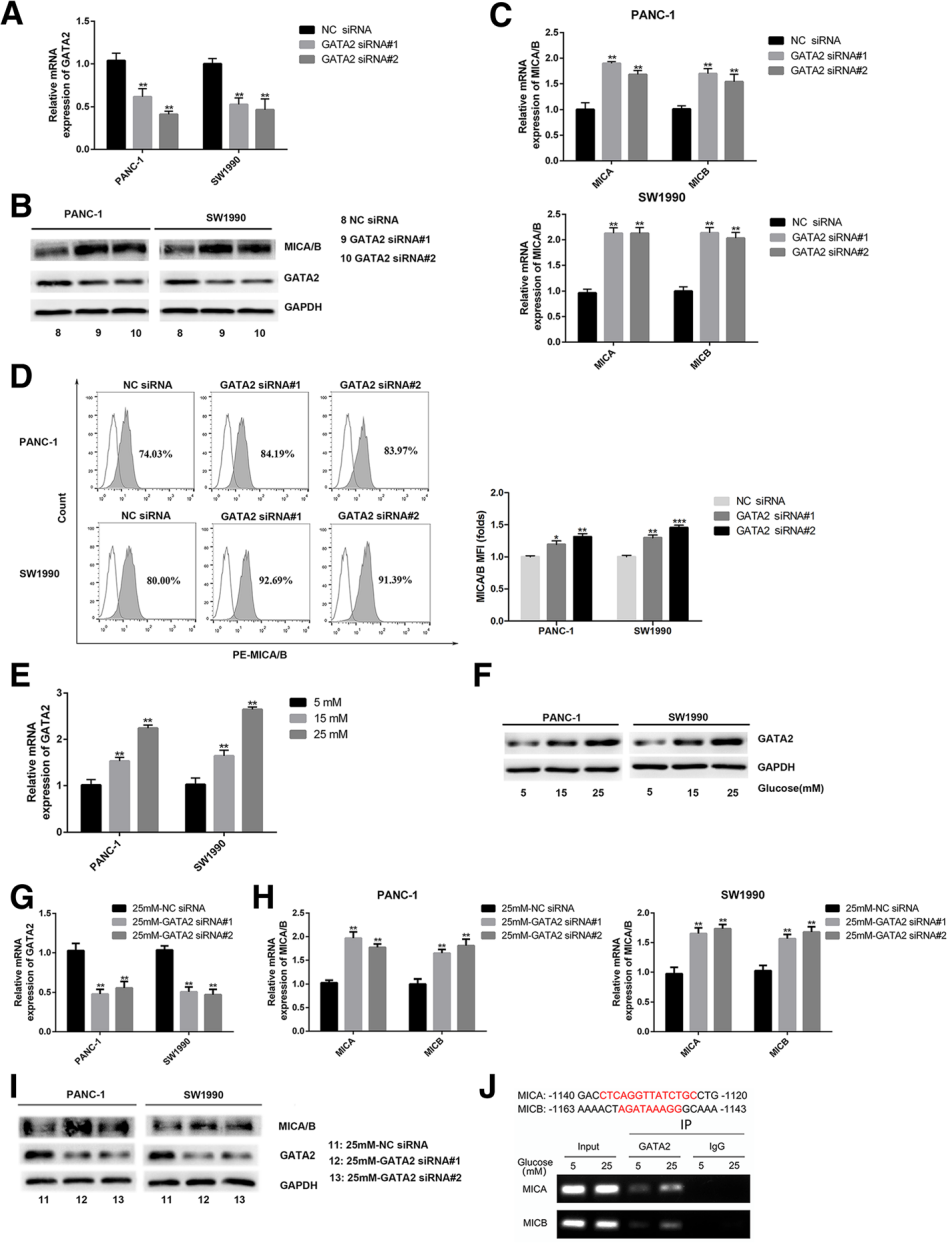
High glucose promotes the expression of GATA2, which inhibits cell surface expression of MICA/B. **a-c** The cell surface expression of MICA/B were measured by Western blot (**b**) and qRT-PCR (**c**) after transfected with GATA2 siRNA (**a**). **d** Representative histograms of flow cytometry demonstrating MICA/B expression in pancreatic cells transfected with GATA2 siRNA. MFI (folds) of MICA/B was evaluated with a Student t test from three independent experiments. **e-f** The expression of GATA2 were detected by qRT-PCR (**e**) and Western blot (**f**) in pancreatic cancer cells treated with high glucose. **g**-**i** The expression of MICA/B were detected by qRT-PCR (H) and Western blot (**i**) after GATA2 siRNA transfection (**g**, **i**) under high glucose environment. **j** The potential site of MICA/B matching the binding sequence of GATA2 is shown in upper panel. The low panel showed that high glucose treatment promoted the binding of GATA2 to the promoters of MICA/B. The graphs shown were representative results of three independent experiments. **P < 0.01

Transcription factor GATA2 has been shown to be involved in tumorigenesis in multiple human tumors, such as the chronic myelogenous leukemia and neuroblastoma [16, 17]. In order to verify the role of GATA2 on MICA/B gene expression, loss-of-function of GATA2 in pancreatic cancer cells was achieved by siRNA knockdown. As shown in Fig. 4a-d, the mRNA and protein level of cell surface MICA/B increased significantly after GATA2 was knocked down by siRNA transfection. We further verified the effect of high glucose on GATA2 expression in pancreatic cancer cells. GATA2 expression was increased with gradual increasing of glucose concentrations, as verified by qRT-PCR and Western blot analysis (Fig. 4e-f). Moreover, expression of MICA/B recovered under hyperglycemic environment after GATA2 knockdown, as detected by qRT-PCR and Western Blot in our experiments (Fig. 4g-i). We further verified that high glucose can promote the GATA2 to bind to the MICA and MICB promoter in pancreatic cancer under high glucose environment using ChIP assay. (Fig. 4j).

**Fig. 5:**
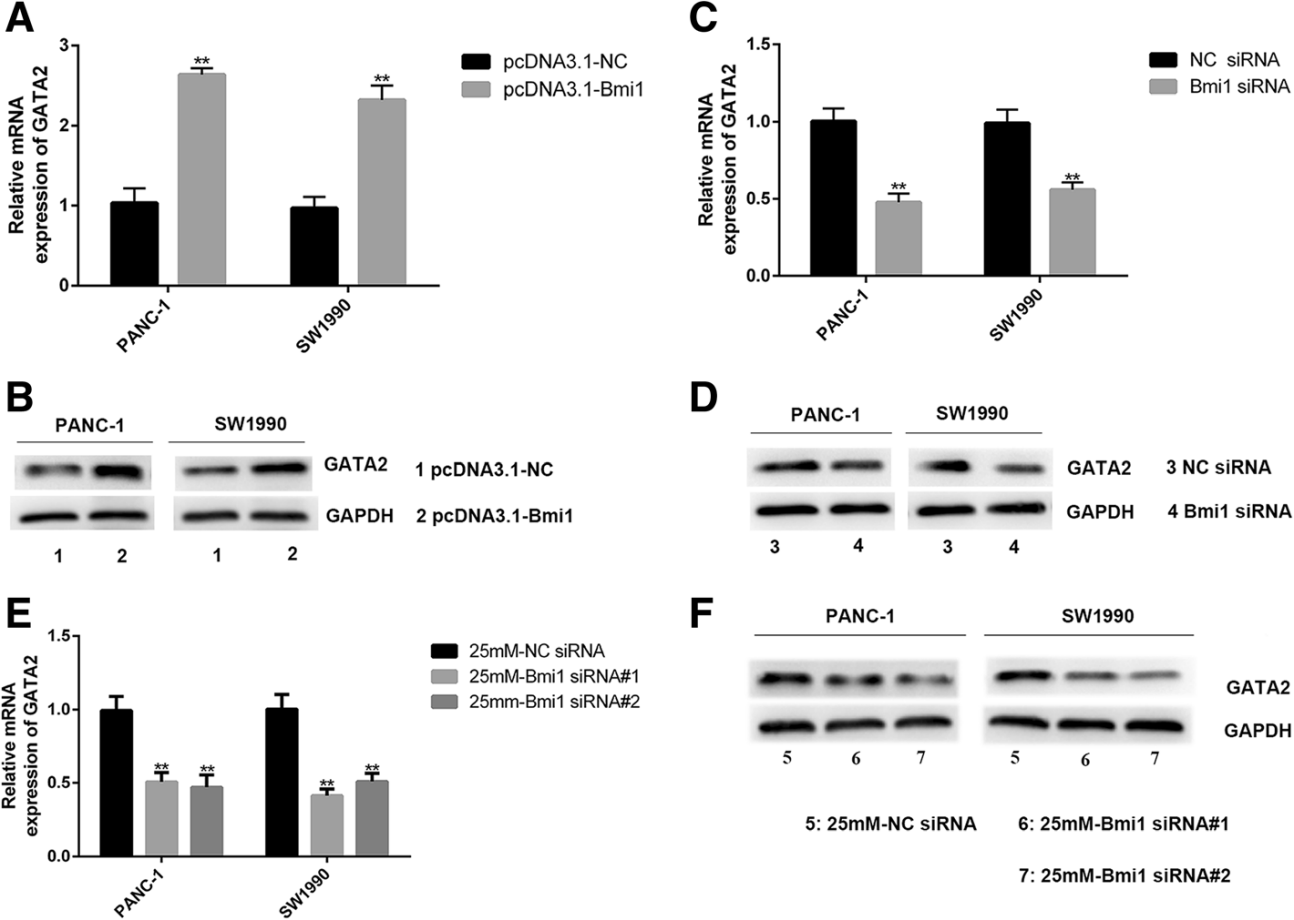
Bmi1 promotes the expression of GATA2 in pancreatic cancer cells. **a-b** qRT-PCR and Western blot analysis of GATA2 expression after transfection with Bmi1 cDNA in PANC-1 and SW1990 cells. **c-d** qRT-PCR and Western blot analysis of GATA2 expression after transfection with Bmi1 siRNA in PANC-1 and SW1990 cells. **e-f** qRT-PCR and Western blot analysis of GATA2 expression after transfection with Bmi1 siRNA under high glucose environment. The graphs shown were representative results of three independent experiments. The data shown were representative results of three independent experiments. **P < 0.01

Next, we investigated whether Bmi1 was involved in regulating GATA2 expression in pancreatic cancer cells. GATA2 up-regulation was verified by qRT-PCR and Western blot after Bmi1 transfection (Fig. 5a-d). Besides, GATA2 expression recovered after Bmi1 was knocked down under high glucose. Our results suggest that Bmi1 inhibits the expression of MICA/B under high glucose through promoting GATA2 expression (Fig. 5e-f).

### High glucose promotes Bmi1 expression through inhibiting AMPK signaling

**Fig. 6:**
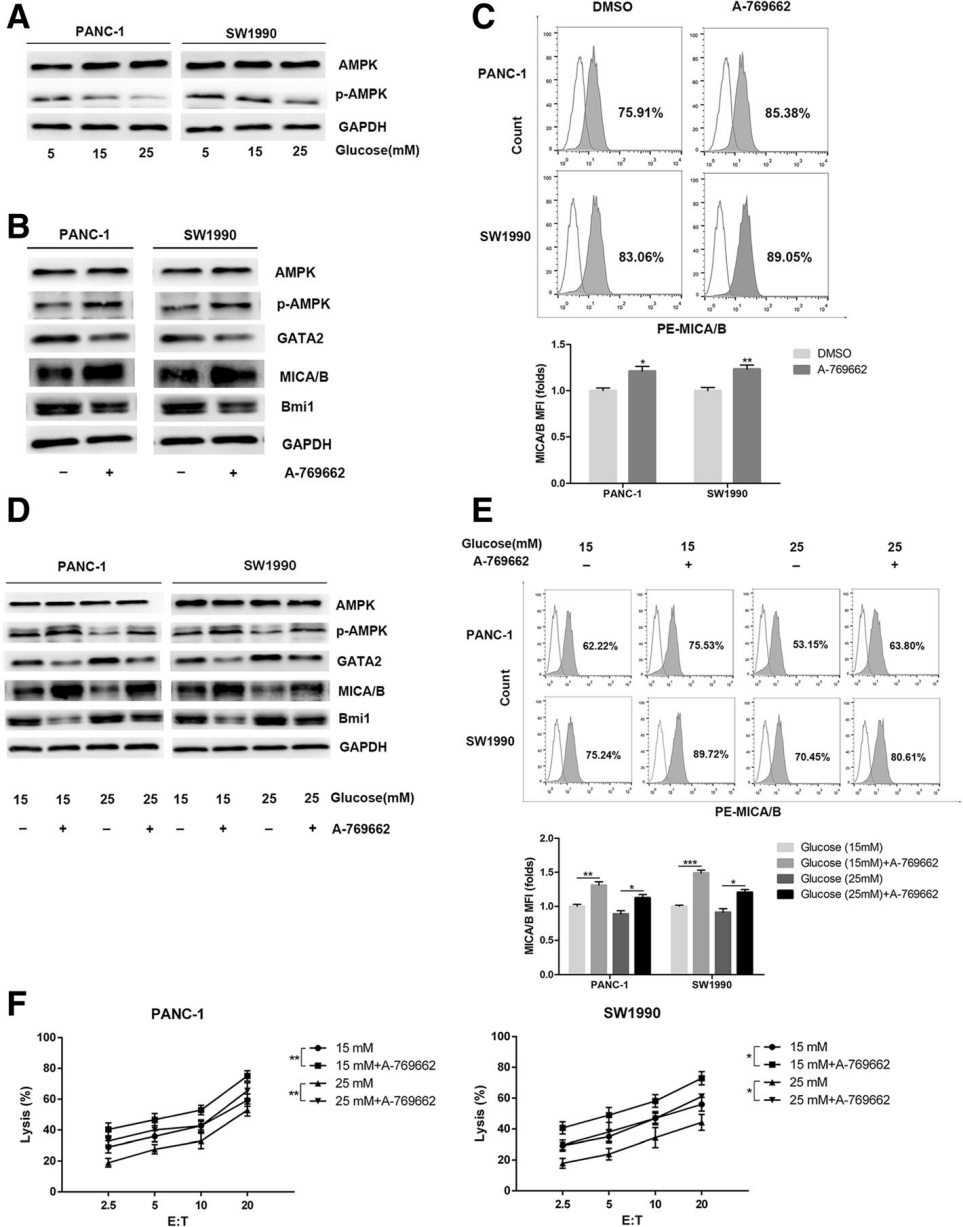
High glucose promotes Bmi1 expression through inhibiting AMPK signaling. **a** Pancreatic cancer cells were treated with different concentrations of glucose for 24 h. Phosphorylation of AMPK was detected with Western blot analysis. **b** The PANC-1 and SW1990 cells were exposed to the AMPK activator A-769662 (20 μM, 2 h) under normal glucose. The expression levels of Bmi1, GATA2 and MICA/B were detected by Western blot. **c** Representative histograms of flow cytometry demonstrating MICA/B expression in pancreatic cells treated with AMPK activator. MFI (folds) of MICA/B was evaluated with a Student t test from three independent experiments. **d** The pancreatic cancer cells were exposed to the AMPK activator A-769662 (20 μM, 2 h) under high glucose. The expression levels of Bmi1, GATA2 and MICA/B were detected by Western blot. **e** Representative histograms of flow cytometry demonstrating MICA/B expression in pancreatic cells treated with A-69662 under high glucose environment. MFI was evaluated with a Student t test from three independent experiments. **f** Effect of AMPK activator on the killing ability of NK cells under high glucose. The graphs shown were representative results of three independent experiments. Data in the graphs represented means ± SD from three parallel experiments. **P < 0.01; *P < 0.05

AMPK plays a vital role in regulating the expression of glucose-dependent metabolism [18]. We further detected the involvement of AMPK signaling pathway in the decreased NK cell cytotoxicity on pancreatic cancer cells induced by high glucose. As a result, high glucose treatment inhibited AMPK activation in pancreatic cancer cells in a dose-dependent manner. The expression of p-AMPK decreased after 24 h of high glucose stimulation (Fig. 6a), as determined by Western blot in comparison with controls. To further verify the role of AMPK signaling pathway in regulating Bmi1 expression, the AMPK activator, A-769662 and AICAR were used to activate AMPK signaling. Treatment with A-769662 or AICAR increased p-AMPK expression, decreased Bmi1 expression, and increased MICA/B expression in pancreatic cancer cells (Fig. 6b-c, Additional file 2: Figure S2). Moreover, after AMPK signaling was activated under high glucose, Bmi1 decreased, GATA2 increased and MICA/B recovered in pancreatic cancer cells (Fig. 6d-e). The killing effect of NK cells on both pancreatic cancer cell lines increased after AMPK activator treatment under high glucose condition (Fig. 6f).

### Hyperglycemia promotes tumor growth and reduces NK cell cytotoxicity in vivo

**Fig. 7:**
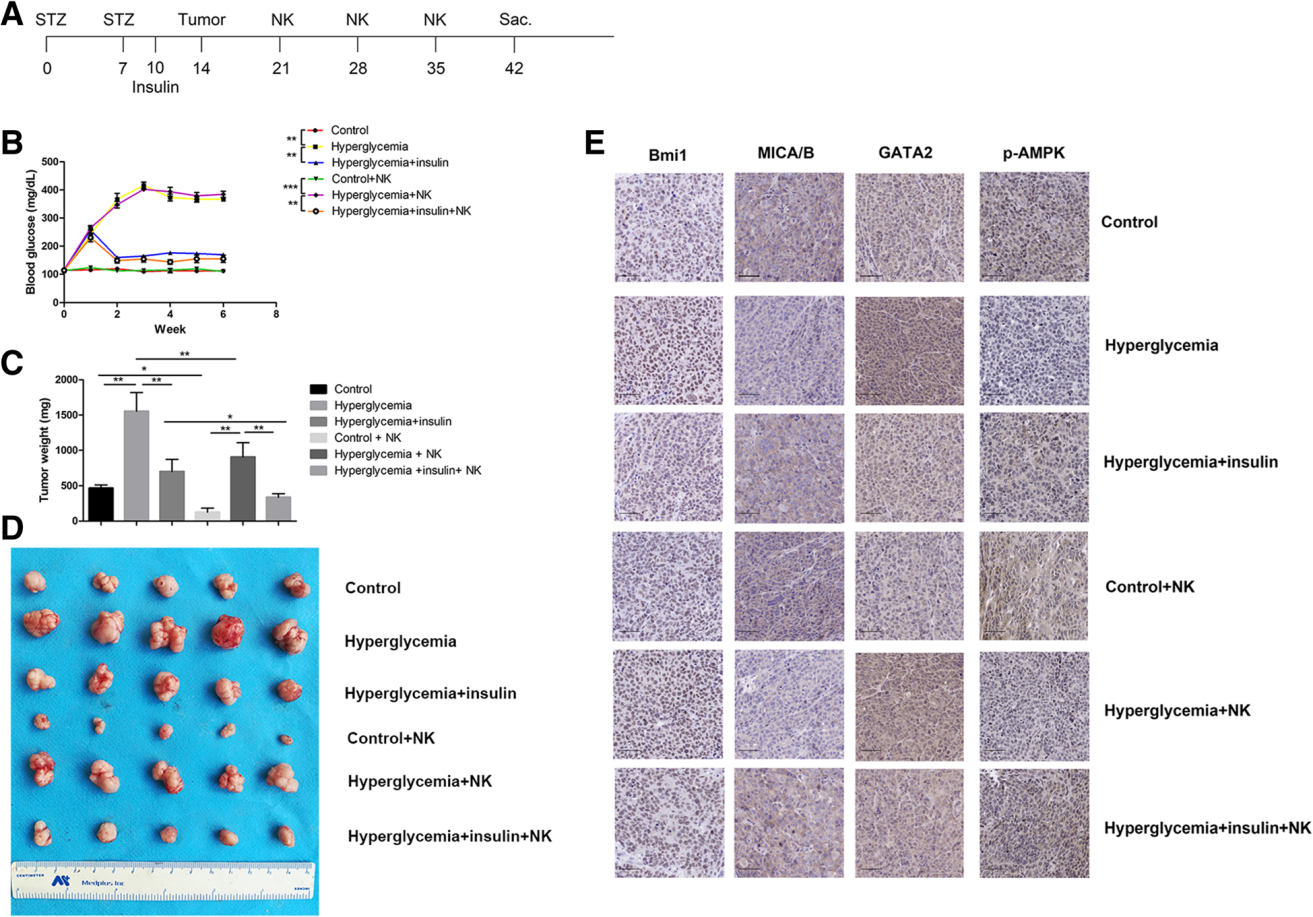
Hyperglycemia promotes tumor growth and reduces NK cell function in vivo*.* **a** The experimental schema of generating diabetes mouse model. **b** The changes in blood glucose after STZ or insulin treatment in the mouse model. **c** The changes in tumor weight after STZ or insulin injection in the mouse model. **d** The changes in tumor volume after STZ or insulin injection in the mouse model. **e** Immunohistochemical staining of Bmi1, MICA/B, GATA2 and p-AMPK in tumor tissues. Data in the graphs represented means ± SD from three parallel experiments. Scale bar, 50 μm, **P < 0.01; *P < 0.05

To confirm the role of hyperglycemia on NK cell killing on pancreatic cancer in vivo, a streptozocin (STZ) induced diabetes mouse model was established in our study (Fig. 7a). In the STZ injected mice, the glucose levels were statistically higher than in controls (Fig. 7b). After the diabetic mice were given insulin, the blood glucose recovered compared to hyperglycemic group (Fig. 7b). We further tested the growth of pancreatic cancer cells in the diabetic mice model. As a result, the tumor growth was significantly increased in the hyperglycemic mice. The tumor size and tumor weight were significant larger in the hyperglycemic mice compared with the control group. (Fig. 7c-d, Additional file 4: Table S2).

We further verify the effect of hyperglycemia on NK cell-mediated cytotoxicity in vivo. After NK cells were injected into the tumor bearing mice, the mice showed significantly reduced tumor volume compared with controls (Fig. 7c-d). While in the hyperglycemic mice, the killing effect of NK cells were significantly weakened. The tumor volume and weight were significantly larger compared with controls. Intriguingly, the function of NK cells recovered when the blood sugar were corrected by insulin injection in hyperglycemia mice. We further determined the expression of Bmi1, MICA/B, GATA2 and p-AMPK in the tumor tissues of different treatment group. As shown in Fig. 7e, hyperglycemia increased Bmi1 and GATA2 level, and decrease MICA/B and p-AMPK in vivo*,* as determined with IHC assessment. These alterations can be reversed when blood sugar was corrected by insulin injection.

## Discussion

Pancreatic cancer is one of the most malignant tumors featured with high mortality. Gene mutation, including K-RAS, TP53, SMAD4, and others, was involved in the molecular pathogenesis of pancreatic cancer [19]. However, these discovered abnormalities to date limitedly contributed to the improvement in therapeutic efficacy or survival among pancreatic cancers patients. The pancreatic cancer has been considered to harbor unique microenvironments. Moreover, pancreatic tumor microenvironments confer highly malignant properties on pancreatic cancer cells and promote pancreatic cancer progression [20]. In this study, we develop our hypothesis that high glucose affects the expression of Bmi1, AMPK, GATA2, and MICA/B and promotes pancreatic cancer cells to escape from immune surveillance. These findings constitute a new signal pathway in response to hyperglycemia, a condition frequently observed in pancreatic cancer patients and are associated with increased mortality and poor survival.

Recent studies suggest that hyperglycemia may play a previously underexplored role in promoting pancreatic cancer progression. Diabetes mellitus has been considered as a potential risk factor for pancreatic cancer and is closely related to the poor prognosis [21, 22]. Accumulating evidences show positive correlation between diabetes mellitus and the increased incidence of cancers [23, 24]. Among the cancers affected by diabetes mellitus, pancreatic cancer exhibits the most obvious correlation with high glucose [5]. Excessive glucose may help cancer cells to maintain their high metabolism and non-controlled proliferation [25]. Moreover, evidence shows that hyperglycemia promotes proliferation and metastasis of pancreatic cancer cells [26]. Multiple mechanisms were involved in the biological association between hyperglycemia and cancer, such as uncontrolled proliferation, hyperinsulinaemia, inflammatory response, et al. [27]. However, there existed sparse literatures regarding the immunological mechanism between hyperglycemia and pancreatic cancer.

In our study, we found that high glucose can inhibit the antitumor immunity by reducing the killing effect of NK cells on pancreatic cancer. This inhibition was related to the reduced MICA/B expression on pancreatic cancer cells. As an important component of NKG2DLs, MICA/B expression is restricted to tumor tissues and plays key roles in mediating the cytotoxicity of NK cells. Decreased MICA/B expression may facilitate cancer immune escape from natural killer (NK) cell-mediated cytotoxicity. Multiple mechanisms have been found to participate in regulating MICA/B expression. It has been reported that DNA damage response pathways, heat shock stress, BCR/ABL oncogene, and bacterial/viral infections can all participate in regulating MICA/B expression [28–31]. In this experiment, we elucidate a new phenomenon that MICA/B can be down-regulated by tumor microenvironment such as high glucose. The mechanism may be one of the tactics that pancreatic cancer escape immune killing. One interesting finding in our study is that high glucose inhibits MICA/B by promoting Bmi1 expression. Abnormal expression of Bmi1 was seen in a variety of cancers, and was related to malignant behaviors of cancer [32, 33]. We previously reported that overexpression of Bmi1 promotes proliferation, malignant transformation, and is related to a poor survival of pancreatic cancer [34]. It has been reported that Bmi1 can enhance the immunomodulatory properties of human mesenchymal stem cells [35]. However, few studies correlated Bmi1 with cancer immune escape, rendering the exploration of Bmi1 in cancer immunity a necessity. In current study, we confirmed that Bmi1 can inhibit anticancer immunity of pancreatic cancer via reducing NK cell killing through suppressing MICA/B expression. We further proved that high glucose can promote Bmi1 expression through inhibiting AMPK signaling pathway. These findings provide new insights of Bmi1 as a central node connecting high glucose and pancreatic cancer development and progression.

In this study, we demonstrated that Bmi1 suppresses MICA/B expression, and this inhibition can be achieved by enhancing GATA2 expression. GATA2 is a member of GATA family transcription factors and contains zinc fingers in its DNA binding domain. GATA2 is involved in the development and differentiation of different types of cells, for example hematopoietic stem cells [36]. Previous study showed that GATA2 was involved in the escape of HBV^+^ HCC cells from NK cell immune surveillance [37]. In this study, we verified that GATA2 can bind to the MICA and MICB promoter and inhibit the transcription of MICA/B genes. Moreover, Bmi1 inhibits MICA/B expression through up-regulating of GATA2 in pancreatic cancer cells, contributing to the immune escape eventually. Our research may open a new avenue to GATA2 research in pancreatic cancer.

Being an abnormal physiological condition in microenvironment, high glucose may affect the biological behavior of cancer cells through changing multiple signaling pathways [38]. We speculate that signaling pathways changed by high glucose may be involved in promoting Bmi1 expression and inhibiting MICA/B expression. AMPK is an important energy sensor which can regulate metabolic or energy homeostasis and participate in almost all aspects of cell function [39, 40]. Moreover, AMPK signaling can be affected by energy metabolism in cells, and its activity was negatively correlated with the invasion ability of tumor cells [41]. In our study, AMPK signaling pathway was inhibited in pancreatic cancer cells treated with high glucose. AMPK inhibition coincides with Bmi1 promotion, GATA2 promotion and MICA/B inhibition. After AMPK was activated, the expression of Bmi1 and GATA2 was inhibited, whereas MICA/B expression was recovered. Importantly, when AMPK signaling was activated, the NK cells could restore its killing effect on pancreatic cancer cells in hyperglycemic environment. Our results suggest that inhibition of AMPK signaling in high glucose can inhibit antitumor immune function by promoting Bmi1 expression and suppressing MICA/B expression. Since AMPK signaling plays a key role in mediating immune escape of pancreatic cancer, it is an ideal target for activating antitumor immunity.

## Conclusion

In summary, this study demonstrates that high glucose can promote pancreatic cancer progression by weakening the killing effects of NK cells on pancreatic cancer. Our finding provides mechanistic insights into the adverse impact of hyperglycemia on pancreatic carcinogenesis and demonstrates the value of targeting AMPK-Bmi1-GATA2-MICA/B axis as potential windows for immunotherapeutic interventions.

## Additional files

Additional file 1: **Figure S1.** Knockdown of Bmi1 increases MICA/B expression and NK cell-mediated lysis in pancreatic cancer cells. A, Knockdown effect of Bmi1 in mRNA and protein levels after transfected with NC-siRNA and Bmi1-siRNA in PANC-1 and SW1990 cell lines. B, C, MICA/B expression in mRNA and protein levels in the presence of Bmi1 knockdown in both pancreatic cancer cell lines. D, The effect of Bmi1 knockdown on the killing ability of NK cells. (TIF 431 kb)

Additional file 2: **Figure S2.** AMPK signaling pathway regulates MICA/B expression in pancreatic cancer cells. **A,** AMPK activator, AICAR (0.5 mM, 6 h), was used to active AMPK singling in PANC-1 and SW1990 cells under normal glucose. The expression levels of Bmi1, GATA2 and MICA/B were detected. **B**, Effect of AICAR on the expression levels of Bmi1, GATA2 and MICA/B under high glucose environment . (TIF 1260 kb)

Additional file 3: **Table S1.** The primers used in qRT-PCR and CHIP analysis. (DOCX 15 kb)

Additional file 4: **Table S2.** The siRNA sequences for GATA2 and Bmi1 knock down. (DOCX 15 kb)

## Abbreviations

AMPK: AMP-activated protein kinase
IHC: Immunohistochemistry
LDH: Lactate dehydrogenase
MFI: Mean fluorescence intensity
MICA/B: MHC class I chain related molecules A/B
NK: Natural killer
qRT-PCR: Quantitative real-time PCR
STZ: Streptozocin
