# Supplementary figures and images for: High glucose promotes pancreatic cancer cells to escape from immune surveillance via AMPK-Bmi1-GATA2-MICA/B pathway
Source: J Exp Clin Cancer Res. 2019 May 14;38:192. doi: 10.1186/s13046-019-1209-9 (PMC6518784; doi:10.1186/s13046-019-1209-9)

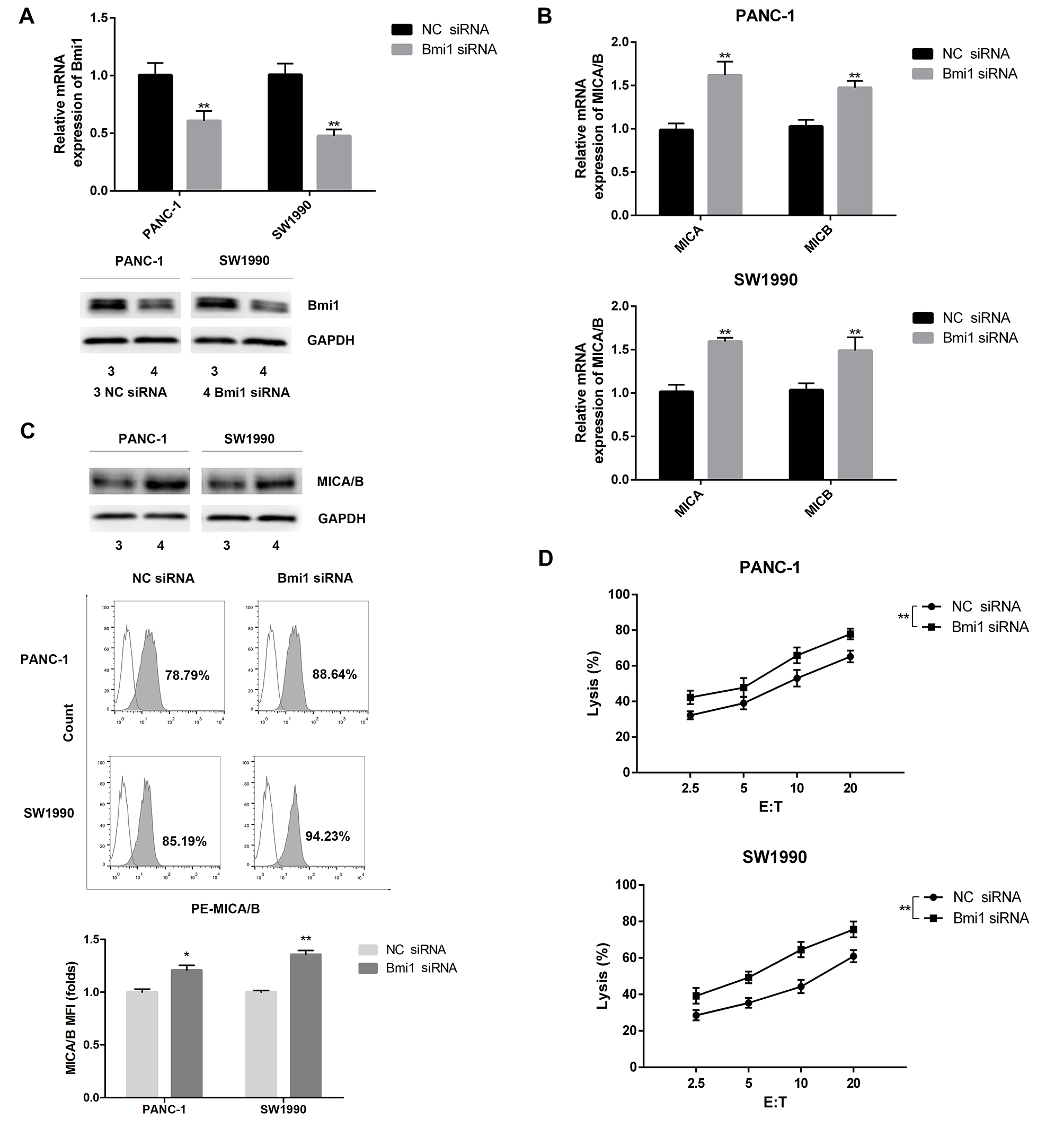

Supplement: Supplementary file 1 — Figure S1. Knockdown of Bmi1 increases MICA/B expression and NK cell-mediated lysis in pancreatic cancer cells. A, Knockdown effect of Bmi1 in mRNA and protein levels after transfected with NC-siRNA and Bmi1-siRNA in PANC-1 and SW1990 cell lines. B, C, MICA/B expression in mRNA and protein levels in the presence of Bmi1 knockdown in both pancreatic cancer cell lines. D, The effect of Bmi1 knockdown on the killing ability of NK cells. (TIF 431 kb) [file 13046_2019_1209_MOESM1_ESM.tif]

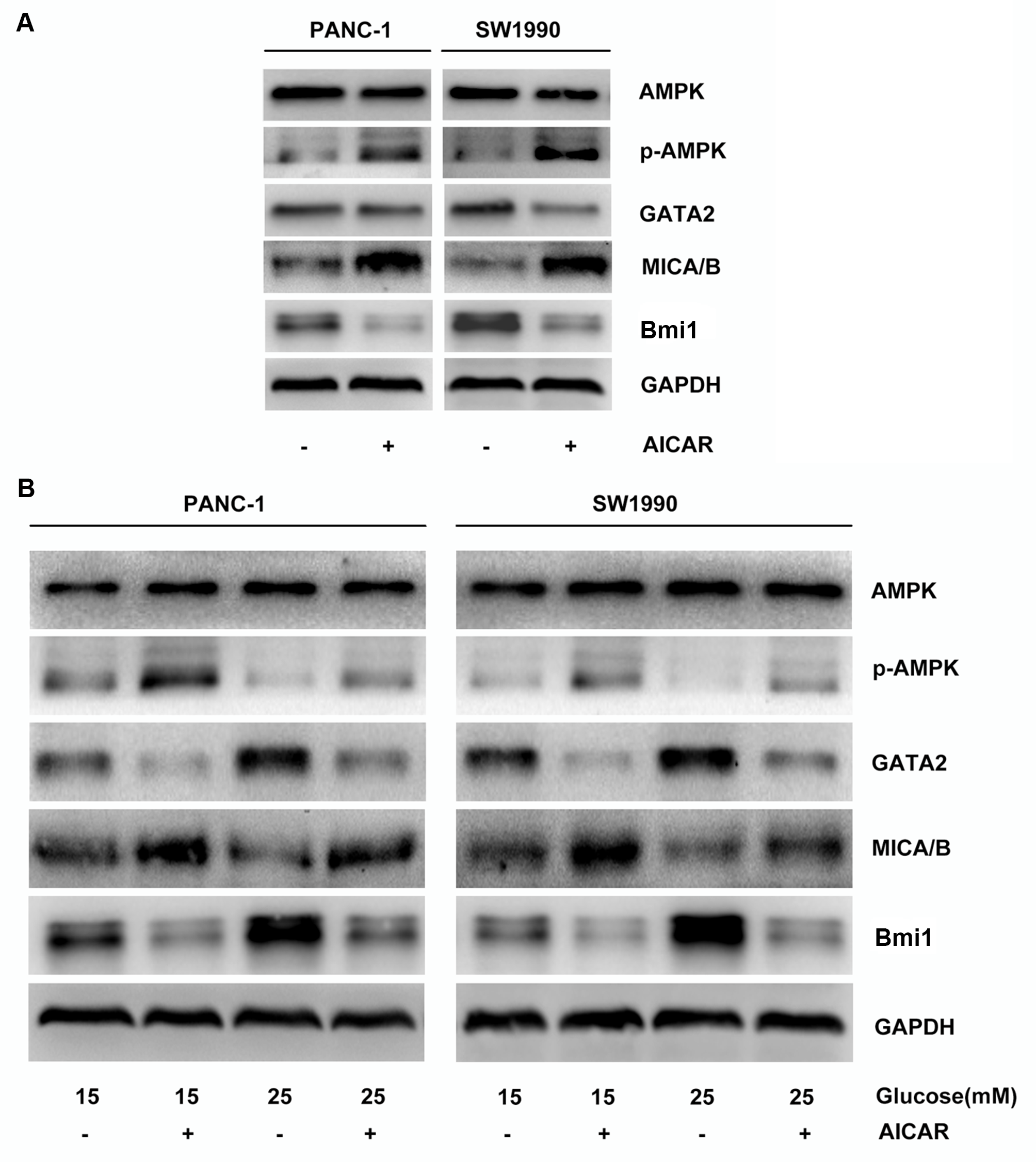

Supplement: Supplementary file 2 — Figure S2. AMPK signaling pathway regulates MICA/B expression in pancreatic cancer cells. A, AMPK activator, AICAR (0.5 mM, 6 h), was used to active AMPK singling in PANC-1 and SW1990 cells under normal glucose. The expression levels of Bmi1, GATA2 and MICA/B were detected. B, Effect of AICAR on the expression levels of Bmi1, GATA2 and MICA/B under high glucose environment . (TIF 1260 kb) [file 13046_2019_1209_MOESM2_ESM.tif]
