# Supplementary material for: High glucose promotes pancreatic cancer cells to escape from immune surveillance via AMPK-Bmi1-GATA2-MICA/B pathway
Source: J Exp Clin Cancer Res. 2019 May 14;38:192. doi: 10.1186/s13046-019-1209-9 (PMC6518784; doi:10.1186/s13046-019-1209-9)
Supplement: Supplementary file 3 — Table S1. The primers used in qRT-PCR and CHIP analysis. (DOCX 15 kb) [file 13046_2019_1209_MOESM3_ESM.docx]

Table S1. The primers used in qRT-PCR and ChIP analysis

| qRT-PCR primer |  | sequence |
| --- | --- | --- |
| GAPDH | forward | 5’- CATGTTCGTCATGGGGTGAACCA -3’ |
|  | reverse | 5’- AGTGATGGCATGGACTGTGGTCAT -3’ |
|  |  |  |
| Bmi1 | forward | 5’- ACAAGACCAGACCACTACT -3’ |
|  | reverse | 5’- TCATTCACCTCCTCCTTAGA -3’ |
|  |  |  |
| MICA | forward | 5’- GACTTGACAGGGAACGGAAA -3’ |
|  | reverse | 5’- CAGGTTTTGGGAGAGGAAGA -3’ |
|  |  |  |
| MICB | forward | 5’- CAGCTACTGGGTCCACTGGT -3’ |
| GATA2 | reverse  forward  reverse | 5’- GTTGGTCATGATCCCTTTGC -3’  5’- GCAACCCCTACTATGCCAACC -3’  5’- CAGTGGCGTCTTGGAGAAG -3’ |

| ChIP-qPCR primer |  | sequence |
| --- | --- | --- |
| MICA | forward | 5’- GCTGGAACTACAGGCACCCA -3’ |
|  | reverse | 5’- GGGCACAGTGGCTCATACCT -3’ |
|  |  |  |
| MICB | forward | 5’- GCTGTCTGCAACTTACCCTCAC -3’ |
|  | reverse | 5’- TGCAGAGAGTGGTGAAAGGAGG -3’ |
