# Supplementary material for: High glucose promotes pancreatic cancer cells to escape from immune surveillance via AMPK-Bmi1-GATA2-MICA/B pathway
Source: J Exp Clin Cancer Res. 2019 May 14;38:192. doi: 10.1186/s13046-019-1209-9 (PMC6518784; doi:10.1186/s13046-019-1209-9)
Supplement: Supplementary file 4 — Table S2. The siRNA sequences for GATA2 and Bmi1 knock down. (DOCX 15 kb) [file 13046_2019_1209_MOESM4_ESM.docx]

Table S2.

The siRNA sequences for GATA2 and Bmi1 knock down

| siRNA | sequence |
| --- | --- |
| GATA2_siR1 | 5’- GAGAGCATGAAGATGGAAA -3’ |
| GATA2_siR2 | 5’- CGGAAGATGTCCAACAAGT -3’ |

| siRNA | sequence |
| --- | --- |
| Bmi1_siR1 | 5’- ATGAAGAGAAGAAGGGATT -3’ |
| Bmi1_siR2 | 5’- CCCGCAGAATAAAACCGAT -3’ |

The individual weight of tumor (mg)

| Control | 506.7 | 414.7 | 493.3 | 417.6 | 498.6 |
| --- | --- | --- | --- | --- | --- |
| Hyperglycemia | 1187. | 1660.5 | 1519. | 1912.9 | 1484. |
| Hyperglycemia +insulin | 617.5 | 608. | 931. | 822.5 | 535.7 |
| Control + NK | 159.5 | 130. | 194. | 95. | 49.2 |
| Hyperglycemia + NK | 852.5 | 1144.4 | 1001.8 | 592.2 | 923.1 |
| Hyperglycemia +insulin+ NK | 391.3 | 355.2 | 275.3 | 296.5 | 373. |
